# Unsupervised Transformer Learning for Rapid and High-Quality MRI Data Acquisition

**DOI:** 10.34133/hds.0340

**Published:** 2025-10-02

**Authors:** Yao Sui, Onur Afacan, Camilo Jaimes, Ali Gholipour, Simon K. Warfield

**Affiliations:** ^1^ National Institute of Health Data Science, Peking University, Beijing, China.; ^2^ Institute of Medical Technology, Peking University, Beijing, China.; ^3^ Institute for Artificial Intelligence, Peking University, Beijing, China.; ^4^ Harvard Medical School, Boston, MA, USA.; ^5^ Boston Children’s Hospital, Boston, MA, USA.; ^6^ Massachusetts General Hospital, Boston, MA, USA.; ^7^ Department of Radiological Sciences, School of Medicine, University of California, Irvine, CA, USA.

## Abstract

**Background:** Magnetic resonance imaging (MRI) is of considerable importance due to its wide range of applications in both scientific research and clinical diagnostics. Acquiring high-quality MRI data is of paramount importance. Super-resolution reconstruction serves as a post-acquisition method capable of improving MRI data quality. Current methods predominantly utilize convolutional neural networks in super-resolution reconstruction. However, convolutional layers have inherent limitations in capturing extensive spatial dependencies due to their localized nature. **Methods:** We developed a new methodology that enables rapid and high-quality MRI data acquisition through a novel super-resolution approach. We proposed an innovative architecture using transformers to exploit long-range spatial dependencies present in images, allowing for an unsupervised learning framework specifically designed for super-resolution tasks tailored to individual subject. We validated our approach using both simulated data and clinical data comprising 40 scans acquired with a 3-T MRI system. **Results:** We obtained images with T2 contrast at an isotropic spatial resolution of 500 μm in just 4 min of imaging time, and simultaneously, the signal-to-noise ratio and contrast-to-noise ratio were improved by 13.23% and 18.45%, respectively, in comparison to current leading super-resolution techniques. **Conclusions:** The results demonstrated that incorporating long-range spatial dependencies substantially improved super-resolution reconstruction, thereby allowing for the acquisition of high-quality MRI data with reduced imaging time.

## Introduction

Magnetic resonance imaging (MRI) holds substantial significance due to its various applications in scientific research and clinical investigations, such as neuroimaging [1–5], functional data analysis [6–10], and healthcare [11–15]. MRI serves as a crucial source of health data that facilitates healthcare management and disease treatment [16–21]. Consequently, obtaining high-quality MRI data is an essential priority. In any MRI setting, spatial resolution, signal-to-noise ratio (SNR), and imaging duration are fundamental factors that determine the quality of MRI data acquisition. Extended imaging sessions can cause stress for patients and increase the likelihood of motion artifacts [22,23]. Therefore, minimizing imaging duration is a crucial objective in MRI operations. On the other hand, acquisitions at high spatial resolution are anticipated to precisely delineate the anatomical structures of the imaged tissues. However, achieving high spatial resolution prolongs the imaging process and reduces SNR.

Many efforts have been made to address the aforementioned challenge. Parallel imaging [24,25] facilitates faster data acquisition while improving the SNR, yet it is restricted by its reliance on specific hardware and platforms. Imaging in ultra-high magnetic fields of 7 Tesla (7 T) and above offers superior SNR; however, imaging at 7 T poses notable risks of vestibulopathy and lacks regulatory approval for children weighing under 66 pounds [26]. As a post-acquisition technique, super-resolution reconstruction has demonstrated the ability to balance spatial resolution, SNR, and imaging duration [27].

Super-resolution reconstruction utilizes multiple low-resolution images as input to produce a single high-resolution image as output. Consequently, this approach is compatible with fast imaging techniques, as low-resolution images can be acquired in shorter scan times. Moreover, the larger voxel size in low-resolution images results in an improved SNR, because each voxel accumulates a greater signal strength while maintaining a constant noise level [28,29]. Research has shown that super-resolution reconstruction is not feasible to enhance in-plane resolution for MRI images acquired via 2-dimensional (2D) stacks or true 3D sequences, primarily due to the frequency encoding scheme [30,31]. However, it proves to be effective in increasing the through-plane resolution [32]. Thus, super-resolution reconstruction necessitates acquiring 2D stacks of MRI image using a large matrix size (high in-plane resolution) with thick slices (low through-plane resolution), with the objective of reducing slice thickness to achieve an isotropic high-resolution reconstruction.

Over the past decade, substantial advances in deep learning have been made, particularly in enhancing MRI super-resolution reconstruction, achieving promising results in the context of scientific research MRI [33–38]. Nonetheless, its application in a clinical setting is not yet feasible. This is largely due to the challenge of obtaining high-quality images as training labels for these advanced models, which need paired low- and high-resolution images from extensive training datasets [39]. Furthermore, the literature indicates that such deep models, trained under these conditions, tend to be unstable when encountering images from different imaging platforms, with varying voxel properties or differing sequences [40,41]. Hence, there is a need to develop a super-resolution model that can be trained solely using patient-specific low-resolution imaging data.

Currently, most super-resolution reconstruction architectures are centered around convolutional neural networks (CNNs) [38,42,43]. However, due to the inherent locality of convolutional layers, these models face challenges in capturing long-range spatial dependencies [44]. Transformers, which incorporate a global self-attention mechanism, have recently gained attention for their potential to address long-range spatial dependency learning [44–47]. Unfortunately, transformer-based architectures generally require substantial training datasets. Acquiring high-resolution images with satisfactory SNRs for use as training labels poses difficulties, primarily due to motion [22]. Consequently, it is crucial to adopt an unsupervised learning approach for transformer-based super-resolution reconstruction. Inspired by the deep image prior (DIP) concept [48], a patient-specific learning framework has recently been developed for super-resolution reconstruction [49]. This method, effectively employing neural network parameterization as a dynamically learned prior, has shown promise in super-resolution reconstruction. However, it relies on CNNs and thus encounters the previously mentioned limitations.

**Fig. 1.**
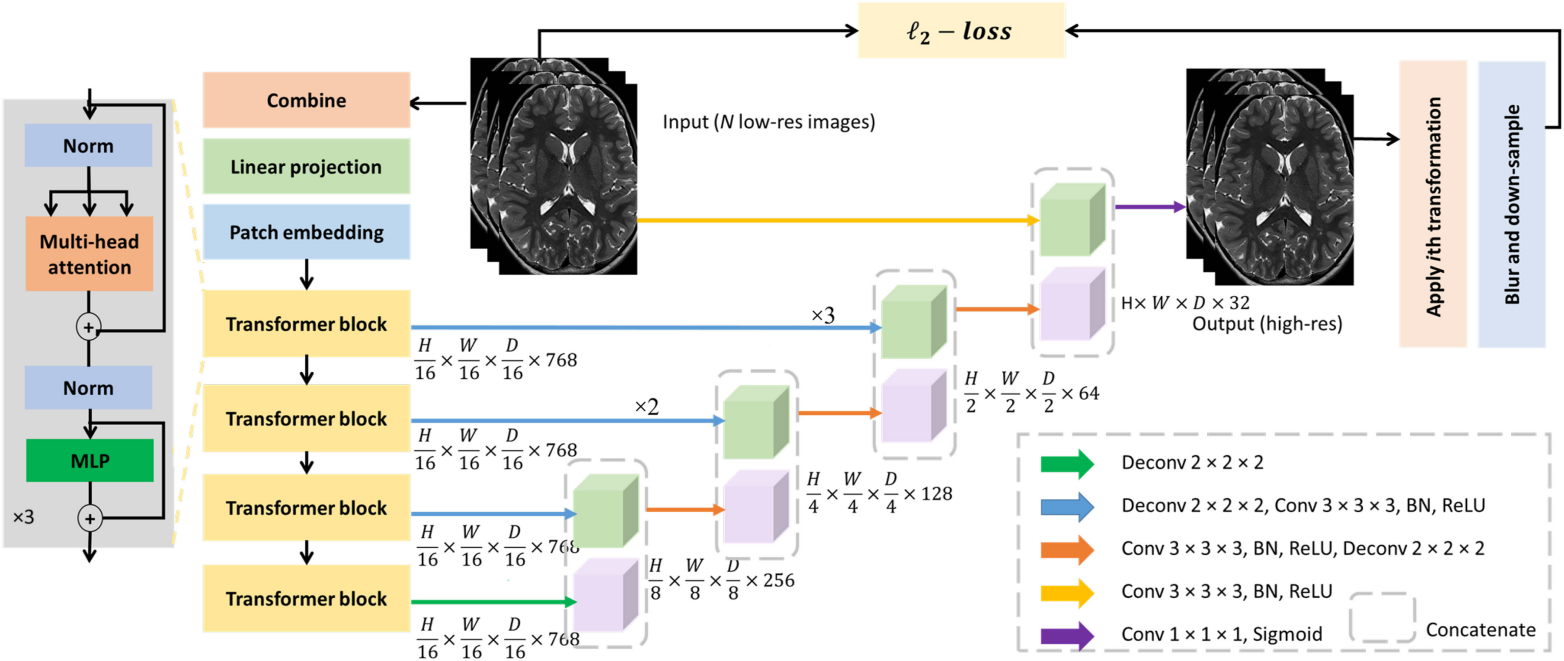
Overview of our approach with an unsupervised transformer learning strategy.

In this study, we introduced a new transformer-based architecture to address the challenge of super-resolution reconstruction. We developed an unsupervised learning strategy for the super-resolution reconstruction, allowing us to train the architecture directly on image data acquired from individual patients. With our proposed super-resolution reconstruction algorithm, we successfully reconstructed high-resolution images with T2 contrast at a 500-μm isotropic resolution in just 4 min of imaging. Figure 1 provides a description of our proposed methodology. We assessed our approach using both simulated data and clinical data consisting of 40 volumetric scans acquired on a 3-T MRI scanner. Our results demonstrated that the ability to learn long-range spatial dependencies substantially improved super-resolution reconstruction. Experimentally, our approach outperformed both direct high-resolution acquisitions and state-of-the-art super-resolution reconstruction methods.

The contributions of this study are summarized below.

• We propose a new methodology that enables rapid and high-quality MRI data acquisition through super-resolution technique. We develop a novel super-resolution approach that utilizes transformers to exploit long-range spatial dependencies present in images, allowing for an unsupervised learning framework tailored to individual subjects.

• We demonstrate that the integration of long-range spatial dependencies significantly improves super-resolution reconstruction. We validate that our approach provides a clinically practical imaging duration for acquiring T2-weighted images with sub-millimeter resolution. Our results indicate that only 4 min of imaging are required to generate an image with T2 contrast and an isotropic spatial resolution of 500 μm, while in parallel, SNR and contrast-to-noise ratio (CNR) are increased by 13.23% and 18.45%, respectively, when compared to state-of-the-art super-resolution techniques.

## Methods

This study seeks to establish an innovative methodology capable of acquiring high-quality MRI images within time frames suitable for clinical application on a patient-specific basis. To accomplish this goal, we devise an unsupervised deep learning technique for super-resolution reconstruction. Our approach encompasses the design of acquisition protocols, modeling of the acquisition process, and the creation of a super-resolution reconstruction algorithm. The subsequent sections present a detailed account of our method.

### Acquisition protocol

The primary goal of this study is to acquire multiple low-resolution images, subsequently reconstructing them into a high-resolution image through a super-resolution reconstruction technique. The time taken for imaging is crucial in obtaining these low-resolution images, especially in the context of clinical applications. Consequently, our acquisition strategy incorporates high in-plane resolution coupled with thick slices. The elevated in-plane resolution helps mitigate the aliasing of Fourier encoding [30,31], while the use of thicker slices facilitates shorter imaging durations and enhanced SNR [50].

Research has indicated that the use of multiple low-resolution images enhances the effectiveness of super-resolution reconstruction compared to relying on a single low-resolution image [51]. Further studies have shown that changing the slice selection direction during the acquisition of these low-resolution images facilitates super-resolution reconstruction [52]. The rationale behind this is that each low-resolution scan contributes high-frequency information along one direction, and super-resolution techniques infer the absent high-frequency details across combined directions. Consequently, our acquisition approach adopts the collection of multiple low-resolution images with various imaging orientations.

In light of the preceding discussion, we develop a T2 turbo spin echo (TSE) sequence specifically for brain imaging on a 3-T MRI system. The imaging protocol specifies an in-plane resolution of 500 μm and a slice thickness of 2 mm. Parameters include a repetition time of 13,100 ms, an echo time of 93 ms, a flip angle of 160°, a bandwidth of 195 Hz/pixel, an echo train length of 16, and an echo spacing of 9.8 ms. We employ GRAPPA parallel imaging [25] with an acceleration factor of 2. Between 60 and 80 slices are acquired in a T2 TSE low-resolution image, depending on the patient’s head sizes. The acquisition time for this T2 TSE image using our method is around 2 min. In comparison, acquiring an image at a 1-mm isotropic resolution using the standard T2 SPACE sequence in clinical settings requires approximately 6 min. This efficiency allows us to collect 3 low-resolution images within a clinically acceptable time frame using our acquisition protocol. In this study, we acquire 2 low-resolution T2 TSE images for each subject, one in the axial plane and the other in the coronal plane, as per our protocol, with the entire imaging procedure taking approximately 4 min.

### Acquisition model

The acquisition model characterizes the imaging process that can be systematically represented in the following steps. A high-resolution observation of the brain undergoes a transformation within its spatial coordinate system due to the varying head positions and orientations across different scans. Subsequently, the transformed image is subjected to blurring effects resulting from slice excitation, as well as phase and frequency encoding. The low-resolution image is then generated by downsampling the blurred image. Hence, the acquisition model is expressed as

$$y_{i}=\left(b_{i}*m_{i}\;\circ \;x\right){↓}_{i}+e,\;i=1,2,\ldots ,N,$$ (1)

where *x* represents the high-resolution reconstruction, $y_{i}$ indicates the *i*th low-resolution image obtained, e signifies the additive noise component, $m_{i}$ denotes the *i*th coordinate transformation, $b_{i}$ corresponds to the *i*th spatial blur kernel, ${↓}_{i}$ refers to the *i*th downsampling operation in the slice selection direction, and ∗ and ∘ denote the convolution and transformation operators, respectively.

We collectively register all low-resolution images to determine the transformation matrices ${\left\{m_{i}\right\}}_{i=1}^{N}$. This registration process relies on mutual information as the similarity criterion, employs a third-order B-spline for interpolation, and uses a Powell optimization method. The spatial blur kernels ${\left\{b_{i}\right\}}_{i=1}^{N}$ are formulated as Gaussian filters, designed to approximate the slice profile for slice excitation. The full width at half maximum of these Gaussian filters is set to match the respective slice thicknesses. These filters are one-dimensional, focusing solely on enhancing through-plane resolution. Their orientations align with the respective directions of slice selection. The downsampling factor is determined by the ratio between through-plane and in-plane resolutions. For practical reasons, the downsampling is performed in the frequency domain rather than via spatial resampling.

### Unsupervised transformer learning

Our goal is to estimate the high-resolution image x from the available low-resolution images $y_{i}$. According to the acquisition model shown in Eq. 1, the noise component *e* is additive and can be effectively approximated by a Gaussian distribution when the SNR is adequate [53]. Thus, the high-resolution image is reconstructed through the inverse problem formulated by the acquisition model:

$$\min_{x}\sum_{i=1}^{N}{\left\|y_{i}-\left(b_{i}*m_{i}\;\circ \;x\right){↓}_{i}\right\|}_{2}^{2}.$$ (2)

Unfortunately, this is an inherent ill-posed problem, as the observations (the number of voxels in $y_{i}$) are substantially fewer than the unknowns (the number of voxels in *x*). Regularization, also known as the prior, is typically incorporated to obtain a high-resolution reconstruction exhibiting the targeted characteristics. Commonly used priors include Tikhonov cost [27], Huber loss [50,54], total variation (TV) [55], bilateral TV [56], low-rank TV [57], edge-preserving potential function [58], and gradient guidance [59,60]. In this study, we incorporate a dynamically learned prior, which is delivered through the parameterization of the transformer network, into the high-resolution reconstruction. Consequently, the super-resolution reconstruction problem is reformulated as

$$\min_{\theta }\sum_{i=1}^{N}{\left\|y_{i}-\left(b_{i}*m_{i}\;\circ \;T_{\theta }\left({\left\{y_{i}\right\}}_{i=1}^{N}\right)\right){↓}_{i}\right\|}_{2}^{2},\;s.t.\;x=T_{\theta }\left({\left\{y_{i}\right\}}_{i=1}^{N}\right).$$ (3)

The transformer network $T_{\theta }\left({\left\{y_{i}\right\}}_{i=1}^{N}\right)$, parameterized by weights θ, processes the entire set of low-resolution images as input. As depicted in Fig. 1, we apply a conventional TV technique [27] to combine these low-resolution images into a single image, which is subsequently resampled onto a high-resolution lattice. The obtained image undergoes tokenization, converting it into image patches of size 16 × 16 × 16 voxels via a linear projection layer. To retain spatial information within these patches, a convolutional layer with a learnable position embedding of dimension 768 is applied. A series of transformer blocks is utilized to exploit the long-range spatial dependencies crucial for high-resolution reconstruction. Each transformer block comprises 12 attention heads alongside a multilayer perceptron of dimension 3,072. Correspondingly, a sequence of deconvolutional blocks is adopted to reconstruct the image across various scales. Skip connections link the transformer to the respective deconvolutional blocks to facilitate reconstruction. To achieve high-resolution reconstruction, we incorporate a 1 × 1 × 1 convolutional layer followed by a sigmoid activation layer. Subsequent degradation of the high-resolution reconstruction is dictated by the acquisition model outlined in Eq. 1, generating all low-resolution images. The discrepancy between the acquired and generated low-resolution images is measured using an $\ell_{2}$-loss. The Adam algorithm [61] is then employed to optimize Eq. 3 concerning the parameters θ, with the process requiring 4,000 iterations at a fixed learning rate of 0.01 to reduce learning error. Our super-resolution reconstruction approach is implemented using PyTorch 1.10.2 [62] and MONAI 0.8.1 [63]. Following the training of the network parameters θ, the high-resolution reconstruction is obtained from

$$x=T_{\theta }\left({\left\{y_{i}\right\}}_{i=1}^{N}\right).$$ (4)

### Experimental setup

We conduct comprehensive experiments to rigorously assess the effectiveness of our proposed method. Through these experiments, we aim to demonstrate the advantages of our approach in super-resolution reconstruction when compared to current leading methods.

#### Datasets

##### Simulated dataset

To validate the accuracy of our proposed approach for super-resolution reconstruction, the inclusion of ground-truth images is essential. Nevertheless, acquiring high-resolution MRI images with adequate SNR poses a substantial challenge, making the acquisition of such high-quality ground-truth images unattainable for validation purposes. As a result, synthetic data are often employed to carry out experiments requiring ground-truth images. The fundamental concept involves using a high-quality image as the ground truth, from which corresponding low-resolution images are generated in accordance with the acquisition model outlined in Eq. 1. This process produces a simulated dataset, enabling a precise assessment of the accuracy of super-resolution reconstruction techniques.

Using the Dryad package [39], we simulated numerous datasets from MPRAGE images with an ultra-high isotropic resolution of 250 μm. To enhance the SNR, the MPRAGE image was formed by averaging 8 separate scans. This process involved a total imaging time of more than 8 h. The resulting ultra-high-resolution MPRAGE image was downsampled to a high-resolution image with a 500-μm isotropic resolution, serving as the ground-truth image. Subsequently, this ground-truth image was further downsampled into lower-resolution images with slice thicknesses of {1, 1.5, 2, 2.5, 3} mm. For each slice thickness, 3 low-resolution images were generated in orthogonal imaging planes. We applied random Gaussian noise to each low-resolution image, with a standard deviation set at 10% of the highest voxel intensity in the image. Ultimately, we reconstructed the high-resolution images using these 3 low-resolution images on each dataset, achieving an isotropic resolution of 500 μm.

##### Phantom dataset

To evaluate the effectiveness of our proposed approach in practical imaging settings, we carried out phantom scans to investigate the image quality provided by our super-resolution reconstruction approach. A benefit of conducting phantom studies is the ability to obtain reference images at high spatial resolution while avoiding motion artifacts; however, a notable drawback is that high-resolution scans can result in a decreased SNR.

An ACR phantom was scanned utilizing a T2 TSE sequence. Three low-resolution images were obtained, each with an in-plane resolution of 1 mm × 1 mm and a slice thickness of 4 mm across the axial, coronal, and sagittal orientations. Additionally, a high-resolution image was acquired at an isotropic resolution of 1 mm, functioning as a reference. The aim of employing this dataset is to reconstruct high-resolution phantom images at an isotropic resolution of 1 mm using these 3 low-resolution images.

##### T2 TSE dataset

To assess practical applicability, it is essential to examine our proposed method within routine clinical scenarios. Therefore, the behavior of all participants during imaging sessions remains unmanageable and unpredictable in this context. In order to evaluate the practical effectiveness of our proposed approach, gold-standard images are required, serving as reference images in each dataset acquired from individual subjects. It should be noted that the overall quality of these reference images might be inferior compared to the images generated through super-solution reconstruction methods. This quality discrepancy arises because direct high-resolution acquisitions suffer from reduced SNR, whereas super-solution reconstruction methods enhance both resolution and SNR simultaneously. This represents the fundamental distinction between the ground-truth images in the simulated datasets and the reference images acquired for the real datasets.

We enrolled 12 volunteers to participate in our study. Using a 3-T Siemens scanner (Siemens Healthcare, Erlangen, Germany), we performed scans on these participants. We instructed them to keep their heads still during the imaging sessions to minimize motion artifacts. A total of 36 T2 image scans were collected for our real datasets. Each participant underwent the acquisition of an axial and a coronal low-resolution T2 TSE image for reconstruction, as well as a high-resolution sagittal T2 SPACE image as a reference image. The reference images possessed an isotropic spatial resolution of 900 μm. The in-plane resolution of the low-resolution T2 TSE images was 500 μm, while the through-plane resolution was 2 mm. Acquiring a low-resolution T2 TSE image took approximately 2 min, whereas the acquisition of a T2 SPACE high-resolution image required about 6 min. All scanning procedures were in compliance with the local institutional review board (IRB) guidelines. We achieved reconstruction of the high-resolution image at an isotropic resolution of 500 μm from the 2 low-resolution T2 TSE images for each dataset.

#### Assessment criteria

The effectiveness of our method was extensively assessed using specific metrics to gauge reconstruction accuracy, SNR, and image sharpness. For the simulated datasets with available ground-truth images, we opted for metrics allowing voxel-wise comparisons (structural similarity index [SSIM] [64]). In contrast, for the real datasets, which exhibit variable contrasts and spatial resolutions across T2 TSE and T2 SPACE images, we utilized metrics that remain unaffected by image contrast and size variations (normalized mutual information [NMI] [65] and Jensen–Shannon divergence [JSD] [66]). In addition, we adopted metrics that do not require a ground-truth or reference image (SNR [29], CNR [29], and sharpness [67]).

##### Structural similarity index

In addition to the mean squared error, the SSIM metric [64] integrates details of image texture when evaluating image quality. This metric necessitates a reference or ground-truth image that matches the size of the image under examination, resulting in a more sophisticated approach for assessing image quality. In this study, considering the resolution discrepancies between the acquired T2 TSE and T2 SPACE images, we registered and resampled the images evaluated on the real datasets to align with the shape and resolution of the reference image.

##### Normalized mutual information

Mutual information serves as a frequently utilized measure to assess the similarity between 2 images, focusing on the probability density of voxel intensities, especially when the images have varying contrasts. A widely recognized variant is the NMI metric [65], which offers quantitative measures standardized within a consistent scale. Notably, the NMI metric does not necessitate alignment of image sizes.

##### Jensen–Shannon divergence

The JSD metric [66] quantifies the divergence between 2 distributions p and q via the expression:

$$d\left(p,\;q\right)=\sum_{i=1}^{N}\left(p_{i}\;\log\frac{2p_{i}}{p_{i}+q_{i}}+q_{i}\;\log\frac{2q_{i}}{p_{i}+q_{i}}\right),$$ (5)

where $p_{i}$ and $q_{i}$ are the *i*th elements of distributions p and q of length N, respectively. In applying JSD to assess image similarity, histograms based on voxel intensity distributions are used to construct p and q. A higher JSD value indicates less similarity between the images. Moreover, JSD computation is solely reliant on histogram data and, thus, does not mandate identical image sizes.

##### Sharpness

The clarity of boundaries between different tissues or lesions in brain MRI images is of paramount importance. A common way to evaluate this characteristic is by assessing the sharpness of image edges. In this study, we utilized the average edge strength (AES) measure [67] to quantify sharpness. AES is calculated as follows:

$$\text{AES}=\frac{1}{\sum_{k}E_{k}}\sqrt{\sum_{k}E_{k}{\left\|G_{k}\right\|}_{2}^{2}}\;,$$ (6)

where $E_{k}$ represents the *k*th voxel derived from the binary edge mask utilizing the Canny edge detector [68], and $G_{k}$ denotes the image gradient at the *k*th voxel, achieved through spatial filtering using Prewitt kernels [69]. An increased AES value signifies sharper image edges. Importantly, the AES measure is applicable directly to the evaluated image and, thus, does not require a reference or ground-truth image. For a thorough evaluation of the improved sharpness provided by our method, we employed a reference image to ensure consistent sharpness measurements. Therefore, we report the normalized sharpness values, which were calculated by dividing the sharpness of a high-resolution reconstruction by that of the corresponding reference image. A sharpness value greater than 1 signifies that the reconstruction is sharper than the reference image, while a value less than 1 indicates reduced sharpness in the reconstruction relative to the reference.

##### Signal-to-noise ratio

SNR substantially influences the quality of MRI images. For the computation of SNR values, we followed the methodology outlined in Ref. [29], applying the formula

$$\text{SNR}=\frac{s}{\sigma },$$ (7)

where s denotes the mean signal strength and σ represents the noise intensity. Segmentation of the evaluated image into gray matter, white matter, and cerebrospinal fluid (CSF) was performed with the FAST toolkit [70] from the FSL suite [71], after skull removal using the BET toolbox [72]. We determined the mean signal strength by taking the average of the voxel intensities of gray and white matter, weighted by their voxel counts. The noise intensity was quantified by calculating the standard deviation of voxel intensities within the background regions.

##### Contrast-to-noise ratio

CNR emphasizes the distinction between various tissue types within the imaging context. To evaluate brain MRI images, we determined the CNR between gray matter and white matter. Adopting the approach delineated in [29], the CNR was evaluated by the expression

$$\text{CNR}=\frac{\left|s_{\text{GM}}-s_{\text{WM}}\right|}{\sigma },$$ (8)

where $s_{\text{GM}}$ denotes the mean signal strength of gray matter and $s_{\text{WM}}$ refers to that of white matter, and σ signifies the noise intensity. Employing an analogous method, we determined the CNR values using the techniques that aid in SNR computations.

#### Baseline methods

Given that our learning approach is unsupervised, it eliminates the need for extensive auxiliary training datasets to train the network. As a result, we did not perform comparisons with supervised methods, but instead focused on evaluating against methods that utilize unsupervised frameworks exclusively. To this end, we incorporated 2 baseline methods in our study.

##### SSGNN

The SSGNN method employs an unsupervised learning framework to produce an enhanced super-resolution reconstruction model utilizing CNNs [49]. This evaluation primarily arises from comparing the SSGNN method with our approach, highlighting the varying impacts of long-range spatial dependency learning via the transformer network versus locality learning through CNNs. For the SSGNN method, we adhered to the hyperparameter settings suggested in Ref. [49].

##### TV

Methods based on TV regularization are extensively employed in super-resolution reconstruction tasks [27]. In our approach, we utilized a standard TV method [27] to offer an initial reconstruction, serving as the input image for the transformer network. Consequently, the disparities observed in the reconstructed images between our super-resolution reconstruction and the TV method demonstrated the advancements afforded by our transformer network learning technique.

## Results

Our super-resolution reconstruction method was executed on an NVIDIA Quadro RTX 8,000 GPU, achieving the reconstruction of a high-resolution image with an isotropic resolution of 500 μm in under 3 h. In the report detailing our experimental results, we termed our method Reconformer to emphasize its use of transformer learning for the purpose of reconstruction.

### On simulated datasets

**Fig. 2.**
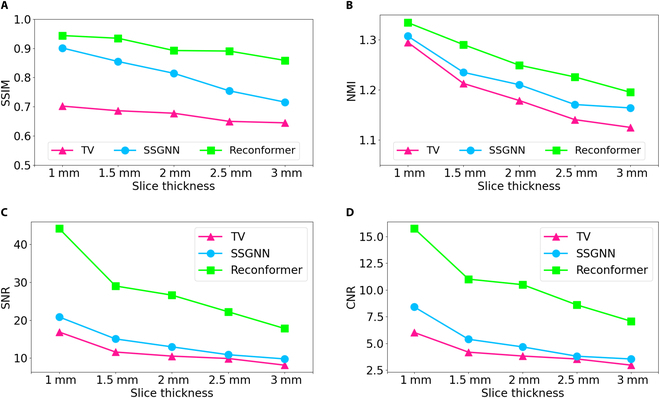
Quantitative comparisons on the simulated datasets at different upscale factors. Our approach (Reconformer) outperformed the 2 baseline methods consistently at all upscale factors. This was evidenced by the superior values in terms of (A) SSIM (↑), (B) NMI (↑), (C) SNR (↑), and (D) CNR (↑). In this context, (↑) indicates that higher values are deemed more favorable.

Figure 2 illustrates the evaluation results of our technique on the simulated datasets across various upscale factors, as compared to those from 2 baseline models. Our method consistently exhibited better performance than the TV and SSGNN methods for all upscale factors when evaluated by SSIM, NMI, SNR, and CNR metrics. The enhanced SSIM and NMI scores indicate that our method achieved higher precision in high-resolution reconstructions on the simulated datasets. The elevated SNR and CNR metrics imply that our method produced higher-quality reconstructed images. This superiority is primarily due to the long-range spatial dependency learning facilitated by our unsupervised transformer learning technique.

**Fig. 3.**
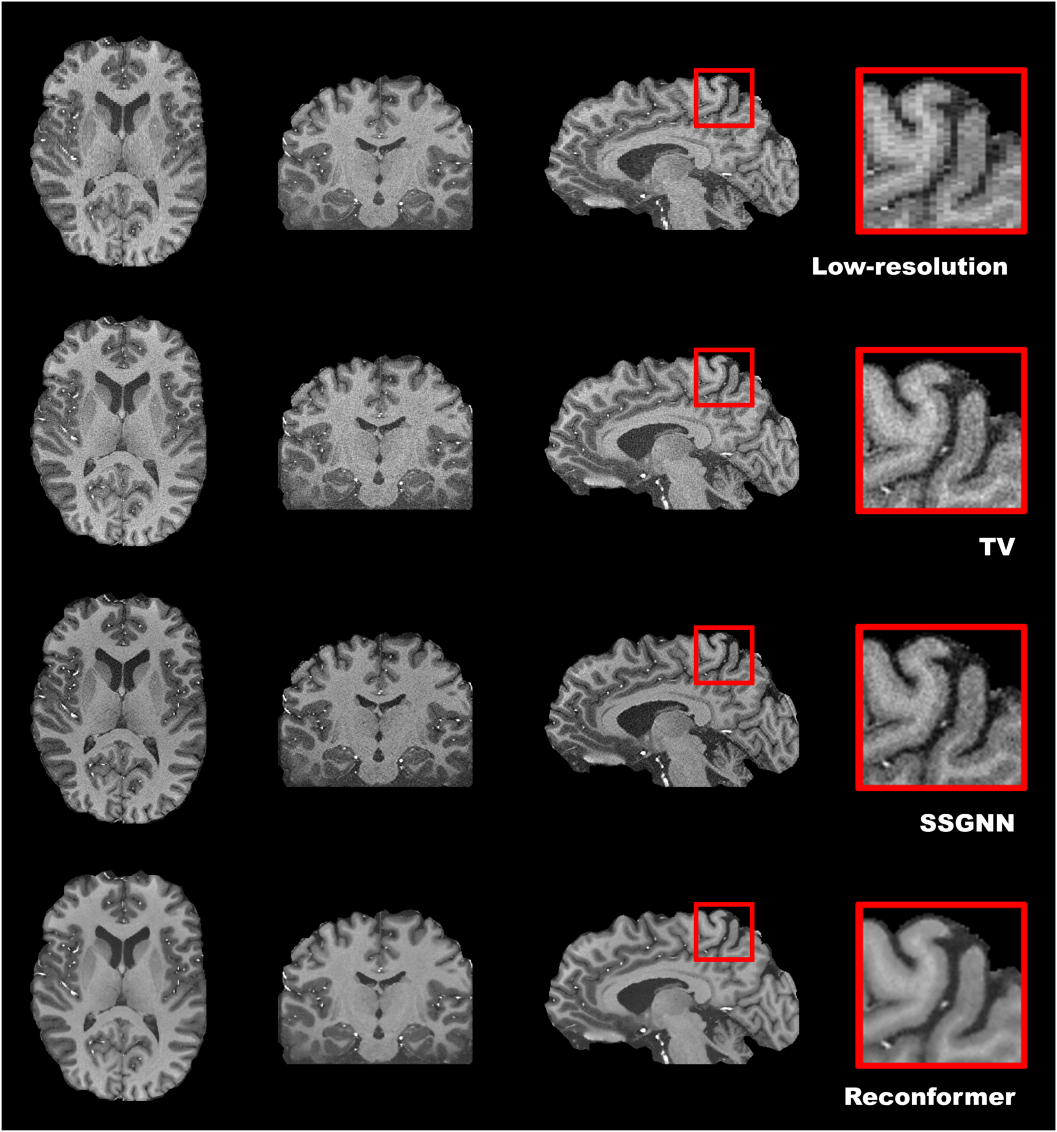
Qualitative comparisons on the simulated datasets. The rows, arranged from top to bottom, depict low-resolution scans alongside the corresponding high-resolution reconstructions obtained via TV, SSGNN, and Reconformer methods, respectively. These images are presented in the axial, coronal, and sagittal planes from left to right. The expanded sections on the far right provide detailed views of the areas highlighted by red boxes to facilitate enhanced visualization. The qualitative analysis indicates that our approach, the Reconformer, successfully produced high-resolution images characterized by superior edge sharpness, improved SNR, and enhanced contrast between gray and white matter in comparison to direct acquisition and the 2 baseline techniques.

Figure 3 presents a qualitative comparison of the simulated datasets. The rows, arranged from top to bottom, depict low-resolution scans followed by high-resolution reconstructions employing the TV, SSGNN, and Reconformer methods, respectively. These images are organized from left to right across the axial, coronal, and sagittal planes. The rightmost boxes provide an enlarged view of the areas delineated by red boxes to facilitate enhanced visualization. The qualitative results demonstrate that our Reconformer method yielded high-resolution images characterized by enhanced edge sharpness, improved SNR, and increased contrast between gray matter and white matter, when compared to direct acquisition and the 2 baseline techniques. As indicated by the quantitative evaluation of SNR in Fig. 2, the reconstructed images produced by the TV and SSGNN methods exhibited substantial noise. In contrast, our reconstructions efficiently mitigated noise, thereby providing more distinct tissue boundaries.

**Fig. 4.**
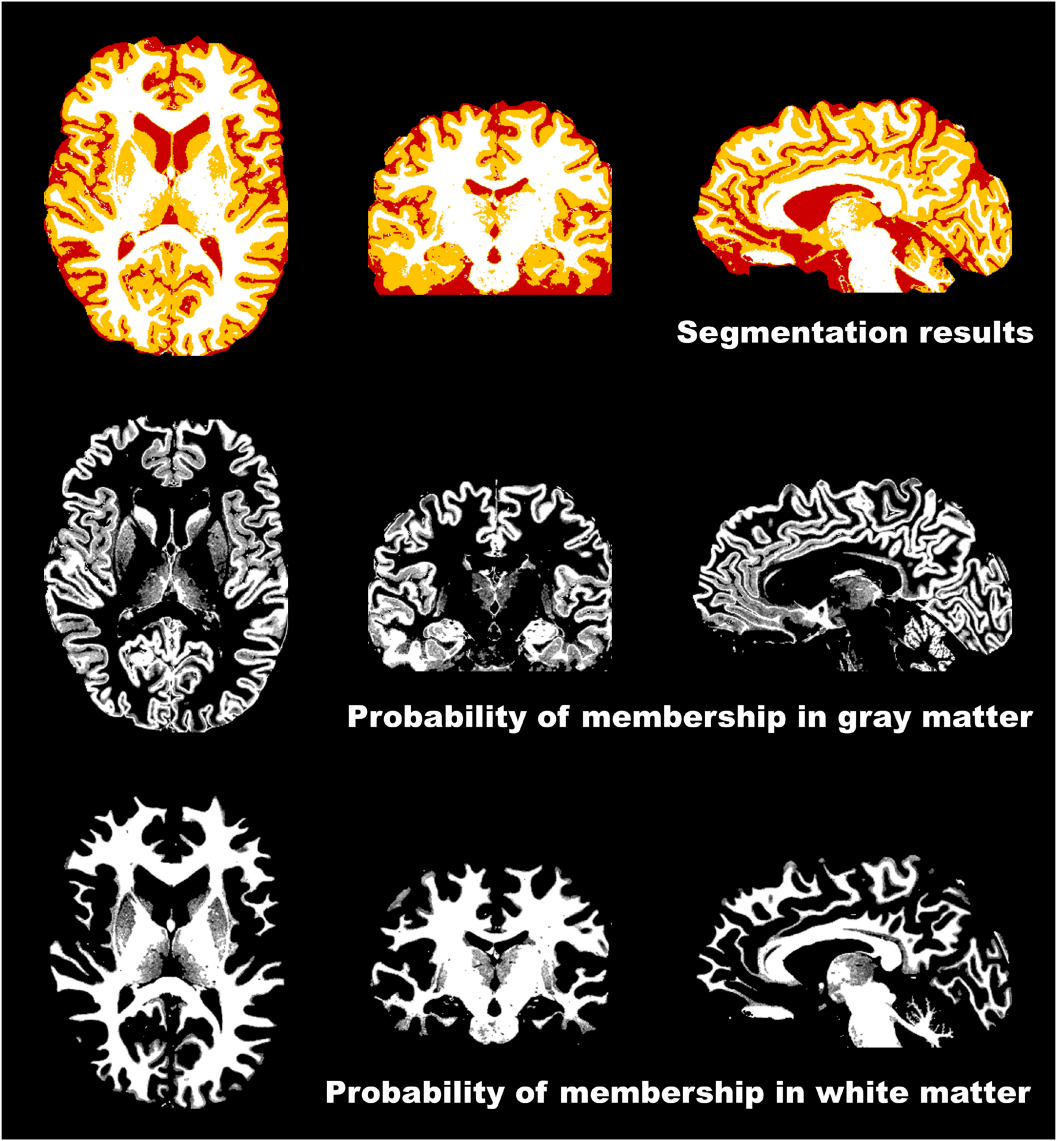
Segmentation of brain tissues on the simulated datasets. The top panel illustrates the segmentation outputs, distinguishing gray matter, white matter, and cerebrospinal fluid (CSF) through various colorizations. The middle and bottom panels depict the segmented gray matter and white matter, respectively, with voxel intensity values extending from 0 to 1, indicating the probability of membership in the respective segmented tissues. It is important to highlight that the inclusion of certain tissues, such as the cerebellum and brain stem, in either the gray matter or white matter categories is inconsequential, since the segmentation was primarily employed for calculating SNR and CNR, rather than for precise morphological assessment.

Figure 4 shows the results of the segmentation of brain tissues on the simulated datasets, used for the SNR and CNR calculations in the quantitative evaluations. The top panel illustrates the segmentation outputs, distinguishing gray matter, white matter, and CSF through various colorizations. The middle and bottom panels depict the segmented gray matter and white matter, respectively, with voxel intensity values extending from 0 to 1, indicating the probability of membership in the respective segmented tissues. It is important to note that the classification of certain tissues, such as the cerebellum and brain stem, within gray matter or white matter categories is deemed nonessential, as the segmentation primarily serves the purpose of SNR and CNR computation rather than exact morphological analysis.

In summary, our Reconformer method exhibited strong performance when tested on the simulated datasets, showcasing its advantages over the 2 baseline techniques.

### On phantom datasets

**Fig. 5.**
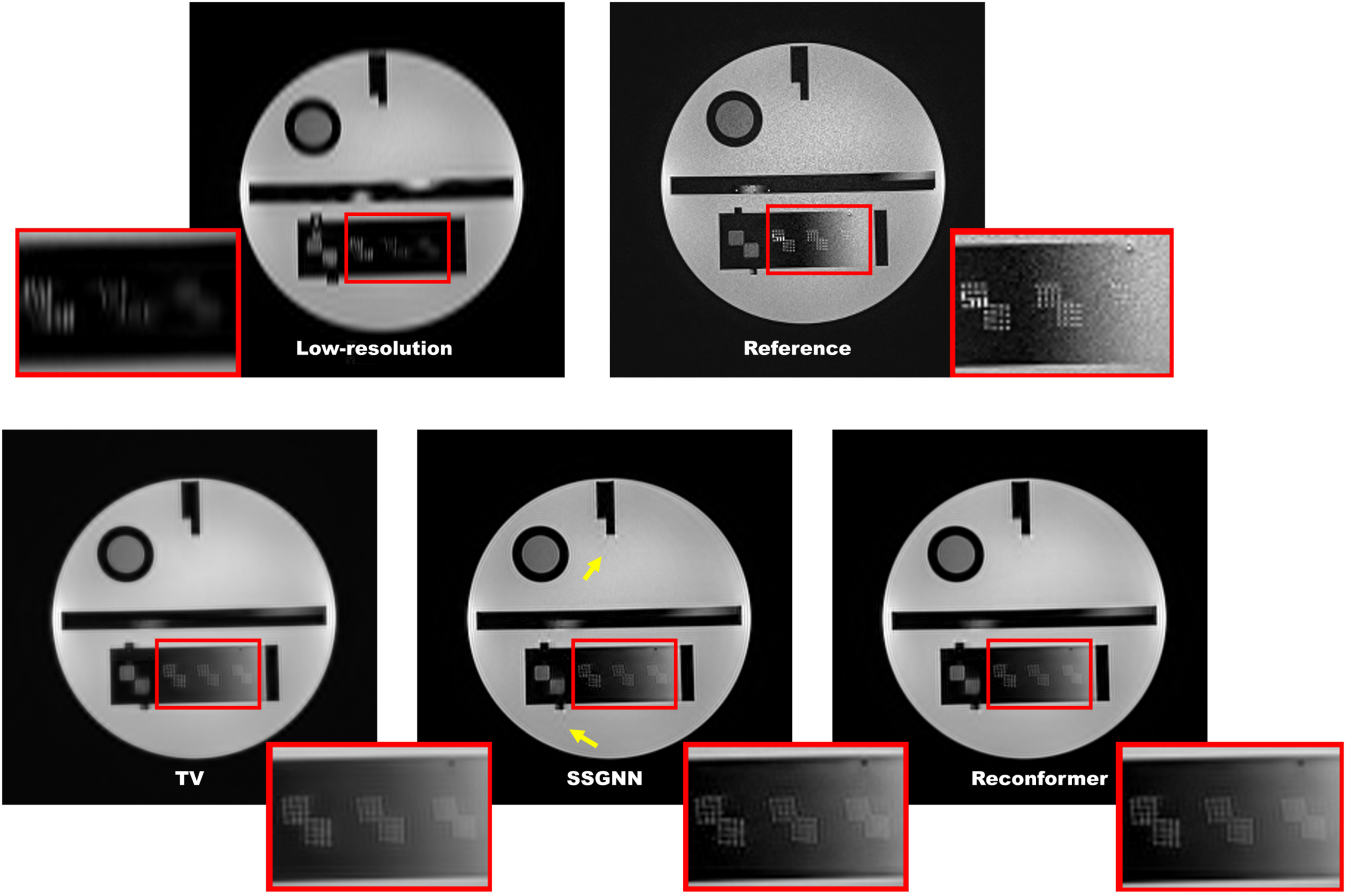
Qualitative comparisons on the phantom datasets. Our method evidently achieved the highest-quality image. In contrast, the reference image showed considerable noise, the TV approach led to excessive smoothing, and SSGNN resulted in artifacts, as indicated by the yellow arrows.

Table 1 presents the quantitative analysis of our approach on the phantom datasets. The evaluation results indicate that our approach, Reconformer, outperformed the baseline methods—specifically TV and SSGNN—across several metrics: NMI (↑), JSD (↓), sharpness (↑), and SNR (↑). Here, an upward arrow (↑) denotes that a higher value is preferred, while a downward arrow (↓) signifies that a lower value is advantageous. Notably, all the super-resolution reconstruction techniques considerably enhanced SNR compared to the reference obtained through direct high-resolution acquisition. Remarkably, our approach improved the SNR by 38.6% relative to the reference. Figure 5 illustrates the qualitative comparisons among the low-resolution image, the reference image, and the high-resolution images reconstructed using TV, SSGNN, and our method. In general, all super-resolution techniques promoted image quality notably by enhancing spatial resolution and reducing noise relative to the low-resolution image. Our method achieved the highest-quality image. The reference image exhibited substantial noise as a result of the high-resolution scan decreasing the pixel size, which reduced the signal intensity in each pixel whereas the noise intensity remained constant. The TV method resulted in an oversmoothed image, whereas SSGNN introduced artifacts, as marked by the yellow arrows.

**Table 1.** Quantitative evaluations on the phantom datasets, measured using NMI, JSD, sharpness, and SNR. The results indicate that our proposed approach, Reconformer, surpassed the baseline methods, TV and SSGNN, across all metrics on the phantom datasets. The most favorable results, highlighted by the relevant metrics, are emphasized in bold type.

Metric	TV	SSGNN	Reconformer (ours)	Reference
*NMI* (↑)	1.138	1.143	**1.147**	-
*JSD* (↓)	0.685	0.581	**0.561**	-
*Sharpness* (↑)	1.032	1.068	**1.074**	-
*SNR* (↑)	26.935	27.638	**28.724**	20.718

### On T2 TSE datasets

**Fig. 6.**
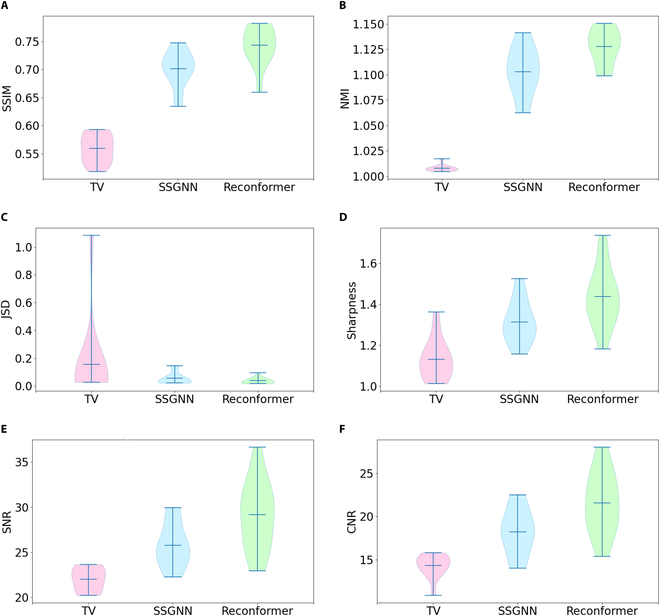
Quantitative comparisons on the T2 TSE datasets. Violin plots were utilized to illustrate the statistical characteristics of the results. Each plot depicts the minimum, mean, maximum, and the rotated empirical distribution of the metrics on either side. The results reveal that our method, Reconformer, substantially surpassed the baseline techniques, TV and SSGNN, regarding (A) SSIM (↑), (B) NMI (↑), (C) JSD (↓), (D) Sharpness (↑), (E) SNR (↑), and (F) CNR (↑). Here, (↑) denotes that higher values are preferable, while (↓) signifies that lower values are favorable.

Figure 6 presents the quantitative comparisons on the T2 TSE datasets. Violin plots [73] were employed to convey the statistical characteristics of the results. Each plot illustrates the minimum, mean, maximum, and the rotated empirical distribution of the metrics on either side. The results indicate that our method, Reconformer, substantially outperformed the baseline techniques, TV and SSGNN, with respect to SSIM (↑), NMI (↑), JSD (↓), sharpness (↑), SNR (↑), and CNR (↑). In this context, (↑) denotes that higher values are more desirable, whereas (↓) implies that lower values are advantageous. Table 2 presents a comprehensive quantitative evaluation on the T2 TSE datasets, using metrics including SSIM, NMI, JSD, sharpness, SNR, and CNR, and providing mean and standard deviation values. Metrics indicating optimal results are accentuated with numerals in bold font. The analysis demonstrates that our proposed approach, Reconformer, consistently surpassed the baseline methods, TV and SSGNN, in all metrics on the T2 TSE datasets. Furthermore, all 3 reconstruction methodologies, TV, SSGNN, and Reconformer, exhibited sharpness scores exceeding 1 for each of their high-resolution reconstructions, signifying an enhanced sharpness relative to the reference scans.

**Table 2.** Quantitative assessment on the T2 TSE datasets, evaluated using SSIM, NMI, JSD, sharpness, SNR, and CNR, yielding mean and standard deviation values. The analysis indicates that our proposed approach, Reconformer, consistently outperformed the baseline methods, TV and SSGNN, across all metrics on the T2 TSE datasets. The best results, as determined by the corresponding metrics, are highlighted by numbers in bold font.

Metric	TV	SSGNN	Reconformer (ours)
*SSIM* (↑)	0.5593 ± 0.0249	0.7014 ± 0.0304	**0.7432** ± 0.0337
*NMI* (↑)	1.0078 ± 0.0317	1.1030 ± 0.0234	**1.1276** ± 0.0167
*JSD* (↑)	0.1555 ± 0.2858	0.0554 ± 0.0406	**0.0389** ± 0.0223
*Sharpness* (↑)	1.1302 ± 0.0971	1.3132 ± 0.1004	**1.4372** ± 0.1419
*SNR* (↑)	22.0243 ± 1.2101	25.7661 ± 2.3579	**29.1738** ± 3.6985
*CNR* (↑)	14.3238 ± 1.3097	18.2133 ± 2.5278	**21.5744** ± 3.4599

Two-sample *t* tests, formulated under the null hypothesis that both datasets originate from independent random samples from normal distributions with identical means and unknown, yet equal, variances, indicated a substantial divergence of our method from the SSGNN approach at a 5% significance level for several metrics. Specifically, the null hypothesis was rejected for SSIM (with $P=5.9\times 10^{-3}$), NMI (with $P=2.5\times 10^{-2}$), sharpness (with $P=2.7\times 10^{-2}$), SNR (with $P=1.7\times 10^{-2}$), and CNR (with $P=1.6\times 10^{-2}$). These results consistently demonstrate that our approach substantially outperformed the second-ranked technique, SSGNN, in these evaluated metrics.

**Fig. 7.**
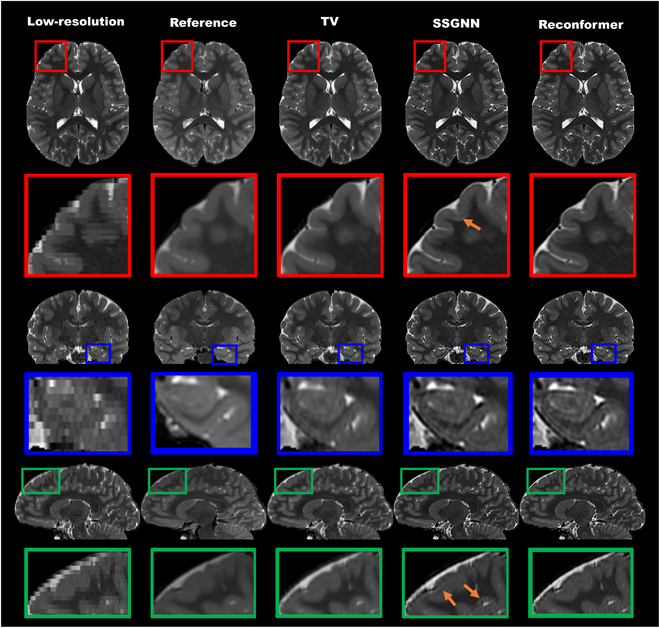
Qualitative comparisons on the T2 TSE datasets. The row sections, arranged from top to bottom, display the axial, coronal, and sagittal slices of a representative subject. In the first 2 columns, the acquired low-resolution T2 TSE and the high-resolution T2 SPACE reference images are shown. The last 3 columns present the high-resolution images reconstructed by TV, SSGNN, and Reconformer, respectively. Among these methods, our approach, Reconformer, demonstrated superior image quality for this subject. In contrast, the reference image and the high-resolution reconstruction using TV exhibit blurred edges and low image contrast, whereas SSGNN introduced subtle artifacts into its high-resolution reconstruction, marked by the orange arrows.

Figure 7 illustrates the qualitative comparisons conducted on the T2 TSE datasets. The sections of the rows, ordered from top to bottom, display the axial, coronal, and sagittal slices of a representative subject. The first 2 columns exhibit the acquired low-resolution T2 TSE and the high-resolution T2 SPACE reference images. Meanwhile, the subsequent 3 columns present the high-resolution images reconstructed utilizing TV, SSGNN, and Reconformer, respectively. Among these methodologies, Reconformer demonstrated superior image quality for this representative subject. In contrast, the reference image and the high-resolution reconstruction employing TV presented blurred edges and diminished image contrast, whereas SSGNN was observed to introduce subtle artifacts into its high-resolution reconstruction, as indicated by the orange arrows.

In summary, when assessed on the T2 TSE datasets, our Reconformer method demonstrated improved image quality relative to direct high-resolution T2 SPACE acquisitions and the 2 baseline techniques.

### Parameter investigation

The size of tokenization patches from the input image is a crucial parameter that affects the performance of our method. Thus, we conducted an analysis to determine the optimal patch size setting in our model. We selected 3 typical patch size values: 8 × 8 × 8, 16 × 16 × 16, and 32 × 32 × 32 pixels. The performance of our approach was assessed using phantom datasets, focusing on reconstruction accuracy (NMI and JSD) and image quality metrics (sharpness and SNR). As shown in Table 3, our approach demonstrated superior performance with a patch size of 16 × 16 × 16 pixels, with respect to both reconstruction accuracy and image quality. This result arises from the observation that smaller patches lack adequate representation capability, while larger patches compromise long-range spatial dependencies. Consequently, we adopted a patch size of 16 × 16 × 16 pixels for all experiments reported in this study.

**Table 3.** Quantitative evaluations on the phantom datasets across various configurations concerning the size of tokenization patches from the input image for our proposed model. This assessment employed metrics such as NMI, JSD, sharpness, and SNR. The results show that our proposed approach, Reconformer, achieved optimal performance with a patch size of 16 × 16 × 16 pixels. The most favorable results, according to the assessed metrics, are emphasized in bold type.

Metric	Patch size		
	8 × 8 × 8	16 × 16 × 16	32 × 32 × 32
*NMI* (↑)	1.140	**1.147**	1.139
*JSD* (↓)	0.577	**0.561**	0.584
*Sharpness* (↑)	1.073	**1.074**	1.073
*SNR* (↑)	27.387	**28.724**	27.037

## Discussion

Our methodology has been extensively assessed via detailed experiments using both simulated and real datasets. This section discusses the study, focusing on its limitations, possible applications, and directions for future exploration.

### Applications

Our super-resolution reconstruction technique facilitates various applications; for example, it aids in the compression of clinical image data to conserve storage space, enhances clinical imaging to improve diagnostic quality, and allows for quantitative imaging while maintaining clinically acceptable scan durations.

The routine clinical use of MRI generates a large amount of images every day. These images must have sufficiently high resolutions to ensure diagnostic precision. Consequently, maintaining and storing the image data presents a substantial challenge. To conserve storage space, it is desirable to minimize the image data size as much as possible. Our super-resolution reconstruction method offers a potential solution to this issue. This method enables the acquisition of only a limited number of low-resolution images per subject and can reconstruct high-resolution images from these low-resolution scans as needed. Thus, with our technique, only low-resolution images need to be stored for each patient, substantially lowering data storage and maintenance costs.

A further direct application of our methodology is in the domain of neonatal brain imaging [3,74–76]. Given the small brain size in newborns, enhanced resolution is essential for imaging. However, increasing resolution alone prolongs the imaging time and reduces the SNR, thus compromising image quality. In addition, due to ongoing myelination, T2 contrast imaging is necessary, which demands extended imaging durations. Our method, as demonstrated, provides a means to balance imaging time, resolution, and SNR, producing high-quality brain images and facilitating neonatal brain imaging.

The literature demonstrates that quantitative MRI methodologies enhance the detection of brain lesions [77–80]. However, evaluating the quantitative features of brain tissues and lesions necessitates multiple MRI scans with different image contrasts. This requirement lengthens scan durations to impractically long periods for clinical applications. Our method enables rapid brain imaging, allowing the acquisition of the requisite number of MRI scans necessary for quantitative assessments of brain tissues and lesions within a clinically feasible scan time.

### Limitations

Super-resolution reconstruction techniques, especially those leveraging deep learning, have frequently faced criticism due to the possibility that the improved resolution could result in visually appealing but artificial enhancements, often referred to as hallucinations. This problem is particularly substantial in the reconstruction of medical images, where maintaining accurate anatomical structures is vital for clinical applications. Our method, distinct from earlier deep learning approaches, depends solely on low-resolution data obtained from an individual patient. By not incorporating insights gained from other subjects, such as through transfer learning with large external datasets, our method reduces the risk of hallucinations. Moreover, employing an explainable model for super-resolution reconstruction can yield reliable results, making it a promising area for future exploration.

## Conclusion

We have developed a new methodology that enables rapid and high-quality MRI data acquisition through a novel super-resolution approach. We have proposed an innovative network architecture grounded in transformers incorporating an unsupervised learning method for super-resolution reconstruction, which facilitates the creation of high-quality images customized for individual subjects. This methodology has allowed us to generate high-resolution images with T2 contrast at an isotropic spatial resolution of 500 μm, requiring merely 4 min of scanning. Our approach has been evaluated using both simulated datasets and clinical data comprising 40 scans using a 3-T MRI scanner. The results demonstrated that incorporating long-range spatial dependencies considerably enhanced the super-resolution reconstruction tasks. The conducted experiments revealed that our technique produced superior results compared to both direct high-resolution imaging and the leading super-resolution reconstruction techniques.

## Ethical Approval

All scans were performed in accordance with the local IRB protocol.

## Data Availability

The datasets used in the simulated experiments are accessible through the URL provided in Ref. [39]. Access to the real datasets is restricted and can be secured upon request.
